# Intraocular Micro-LED Epiretinal Projection for Anterior Segment Blindness: Design and Large-Animal Feasibility Study

**DOI:** 10.3390/bioengineering13040397

**Published:** 2026-03-29

**Authors:** Bingao Zhang, Jiarui Yang, Hong Jiang, Zhiying Gui, Shengyong Xu

**Affiliations:** 1 Key Laboratory for the Physics and Chemistry of Nanodevices, Institute of Physical Electronics, Department of Electronics, Peking University, Beijing 100871, China; zhangbingao@pku.edu.cn; 2 Beijing Key Laboratory of Restoration of Damaged Ocular Nerve, Department of Ophthalmology, Peking University Third Hospital, Beijing 100191, China; 3 Department of Neurobiology, Neuroscience Research Institute, School of Basic Medical Sciences, Peking University, Beijing 100871, China; hong.jiang@hsc.pku.edu.cn (H.J.); zhiyinggui@stu.pku.edu.cn (Z.G.); 4 Key Laboratory for Neuroscience, Ministry of Education/National Health Commission, Peking University, Beijing 100871, China

**Keywords:** intraocular projection, anterior segment blindness, Micro-LED display, epiretinal implant, transscleral implantation, artificial vision

## Abstract

Irreversible anterior segment blindness with preserved retinal integrity (e.g., dense corneal opacity) remains a major clinical challenge because effective sight-restoring options are limited. Here, we describe an intraocular micro-light-emitting diode (Micro-LED) epiretinal microdisplay intended to deliver patterned optical stimulation to intact photoreceptors by bypassing opaque anterior optics. The prototype was based on a color-capable VGA microdisplay (640 × 480 pixels) and operated at <30 mW under typical conditions. An ultra-thin flexible cable and a copper-mesh–reinforced polydimethylsiloxane (PDMS) encapsulation provided a compact, conformable intraocular package with high pixel density. We evaluated a monochromatic (green) prototype in a single beagle eye (n=1) using a transscleral implantation approach and performed 7 days of postoperative follow-up with slit-lamp examination and multimodal imaging. Patterned stimulation via the implanted display elicited flash-evoked visual evoked potentials (VEPs) with consistent within-session waveform morphology, providing preliminary neurophysiological surrogate evidence of upstream visual pathway activation under the tested conditions in this single-animal pilot. The short-term postoperative course included transient hypotony and anterior segment inflammation, and implant rotation with associated inferior retinal detachment was observed by day 7, highlighting current biomechanical limitations. Beyond anterior segment opacity, the same intraocular optical interface could be explored as a modular light-delivery platform to pair with emerging retinal therapies (e.g., optogenetics), pending chronic safety and functional validation. This pilot large-animal study therefore provides a translationally relevant testbed while delineating key engineering constraints that must be addressed next.

## 1. Introduction

Vision is the primary sensory modality through which humans perceive and interact with the world. Yet, millions of people worldwide suffer from irreversible visual impairment or blindness due to severe pathologies or structural damage affecting the anterior segment of the eye—such as the cornea, iris, lens, and vitreous body [1,2]. These conditions, often caused by trauma, infection, congenital anomalies, or immune-mediated diseases, compromise the eye’s natural optical pathway and drastically diminish visual function, even when the retina and optic nerve remain structurally and functionally intact.

Current treatments for anterior segment disorders, like corneal transplantation and intraocular lens (IOL) implantation, face significant limitations in restoring clear vision. Corneal transplantation, though effective for some, is severely hampered by a global shortage of donor tissue, with an estimated 12.7 million patients awaiting transplants worldwide [3]. Even when performed, long-term success is not guaranteed; high-risk cases, such as those with corneal vascularization, see 10-year graft survival rates as low as 35% due to complications like immune rejection and infection [4]. Moreover, transplantation is often impossible or too risky for patients with severe ocular surface diseases (e.g., Stevens–Johnson syndrome) [5,6]. While external aids like scleral lenses offer an alternative for those unsuitable for or failing transplantation, they often prove inadequate or intolerable for severe surface disorders [7,8].

Critically, existing implantable retinal prostheses (e.g., Argus^TM^ II, Alpha IMS/AMS, Prima) are fundamentally mismatched for anterior segment blindness. These devices bypass degenerated photoreceptors by stimulating residual retinal neurons to elicit visual perception [9,10,11,12,13]. Yet they exhibit intrinsic limitations: (1) low physical resolution of electrode arrays (typically ranging from a few dozen to just over one thousand stimulation sites) restricts object recognition and mobility [14]; (2) unnatural phosphene perception induced by ganglion cell stimulation differs drastically from natural vision [15,16]; (3) high maintenance burdens mandate lifelong immunosuppressive drops, monthly clinical visits, and emergency protocols; (4) prohibitive costs (∼$110,000) limit accessibility [17]. More fundamentally, their electrical stimulation paradigm bypasses functional photoreceptors—a suboptimal approach for patients with intact retinae.

Given that many patients with corneal blindness still retain structurally intact retinae, an alternative concept—intraocular projection—has been explored to directly project optical images onto the retina, thereby bypassing anterior segment opacities. Early explorations in the 1990s and early 2000s included Samiy et al.’s design of a miniature electronic display coupled with a focusing element implanted in the anterior eye [18], and Aharoni et al.’s patent describing a similar system integrated within a large intraocular lens [19]. Around the same time, several “intraocular vision aid” prototypes were proposed, incorporating image-processing cameras, low-resolution LED displays, and wireless power/data transmission modules [20,21,22]. However, these efforts were limited by the technology of the era: display resolution was insufficient for functional vision, optical assemblies were bulky, and system integration was incomplete. The only in vivo validation consisted of a single wirelessly powered LED implanted into rabbit eyes, which demonstrated long-term biocompatibility but offered no meaningful visual function [23].

Recent advances in high-resolution LCD, OLED, Micro-LED, and LCOS microdisplays have renewed interest in intraocular projection [24,25,26]. The Stanford group developed early prototypes, including a Kopin VGA LCD system with a theoretical visual acuity of 20/130—better than the threshold for legal blindness but still far from natural vision [27]. More recently, in collaboration with Mojo Vision, they created an intraocular lens–shaped implant embedding a 0.48 mm Micro-LED chip and optics [28]. Implanted in rabbit eyes, the device remained stable for six months, though evaluation was limited to safety without functional validation. A related effort introduced an ultrathin wireless FlexLED implant with 8192 pixels conforming to the retinal surface and combined with optogenetic stimulation of retinal ganglion cells [29]. In photoreceptor-degenerate rabbits under anesthesia, the device evoked localized cortical responses, but behavioral restoration and long-term safety remain unverified. Overall, intraocular projection offers a promising way to bypass anterior segment opacities, yet most efforts remain at the prototype or small-animal stage. Clinical translation is therefore at an early stage. Against this backdrop, our study provides the first large-animal demonstration in dogs, establishing critical feasibility and safety data.

The present study differs from prior intraocular projection work in several respects. Compared with the Stanford VGA LCD prototype [27], the Micro-LED format eliminates backlighting, yielding higher peak luminance and a thinner self-emissive package. Compared with the Mojo Vision intraocular lens–shaped device [28], which replaces the crystalline lens and targets presbyopia, the present epiretinal approach is designed for patients with intact natural optics, requiring no lensectomy. Compared with FlexLED [29], which was validated in optogenetically sensitized photoreceptor-degenerate rabbits, the present device targets the intact photoreceptor pathway without genetic modification and is evaluated here in a large-animal (canine) model whose ocular dimensions more closely approximate the human eye.

**Figure 1:**
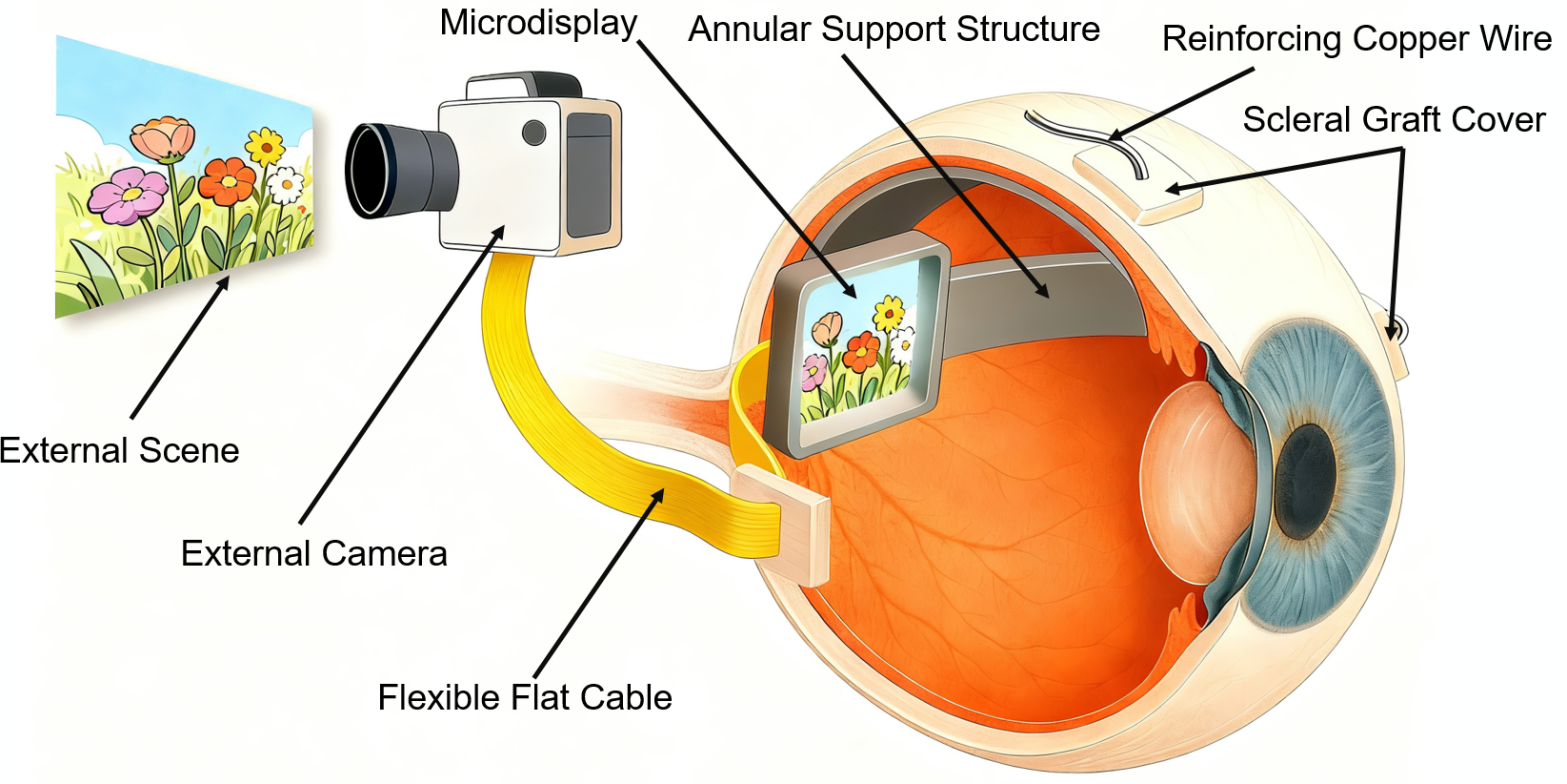
Schematic diagram of the epiretinal visual prosthesis system. An external camera captures visual input and transmits signals via a flexible flat cable (FFC) to an intraocular microdisplay. The microdisplay is stabilized within the vitreous cavity by an annular support structure reinforced with metal wire and covered with a scleral graft. The copper mesh reinforcement is embedded within the support to ensure structural stability and biocompatibility.

To address these unmet needs, we present a conceptual design and a proof-of-concept implementation of a miniaturized, full-color–capable Micro-LED epiretinal microdisplay intended to bypass opaque anterior optics by delivering patterned light to intact retinal photoreceptors (Figure 1). The intraocular device integrates a VGA-resolution Micro-LED array with ultra-thin cabling (150 μm) and reinforced PDMS encapsulation; benchtop characterization indicates high pixel density (640×480), low power consumption (<30 mW), and short-term encapsulation stability. Given that thermal loading is a critical safety concern for intraocular light sources, finite-element bioheat simulations were conducted to assess the steady-state thermal stability and estimate the maximum intraocular temperature rise under continuous operation. In the present work, we then evaluated a monochromatic (green) prototype in vivo using a Transscleral Implantation Technique Covered by a Xenogeneic Scleral patch (TIT-XSP) in a canine model, and assessed short-term device operability, postoperative ocular tolerance, and the functional viability of the optical pathway, as evidenced by stimulus-evoked cortical responses in VEP recordings.

While fundamentally different from electrode-based retinal prostheses that elicit phosphenes via direct electrical stimulation, intraocular optical projection may provide a complementary route for delivering higher-density patterned stimulation and for exploring multicolor operation through physiological phototransduction. Overall, our work provides a pilot large-animal testbed for intraocular optical projection and delineates key engineering and validation challenges that remain. The presented platform may serve as a modular, low-power optical interface that could be further optimized in future studies, including chronic multi-animal evaluation with functional endpoints, as well as potential integration with emerging optogenetic therapies or vision-assistive navigation paradigms, for patients with severe anterior segment opacities and preserved retinal function.

## 2. Materials and Methods

### 2.1. Microdisplay Module and Driver Electronics

A commercially available 0.13-inch VGA Micro-LED microdisplay (JBD013VGA, Jade Bird Display, Shanghai, China) was used as the optical engine of the implant. The device integrates a 640×480 active-matrix array over a 2.64×2.02mm emitting area (pixel pitch 4 μm) within a 16.8×5.1×1.78mm package, providing a compact light source suitable for intraocular use. The green-emitting variant (peak wavelength 525nm) was selected for the present prototype; full-color versions based on the same platform are available for future development.

The microdisplay is driven by a custom control board that receives video input from an external computer via USB or HDMI and transmits synchronized data and bias signals to the implant through an ultra-thin flexible flat cable (FFC). Under typical operating conditions, the module delivers sufficient luminance for epiretinal projection with a total electrical power consumption of approximately 30mW and supports real-time image presentation with grayscale and refresh rates adequate for pattern stimulation experiments.

**Figure 2:**
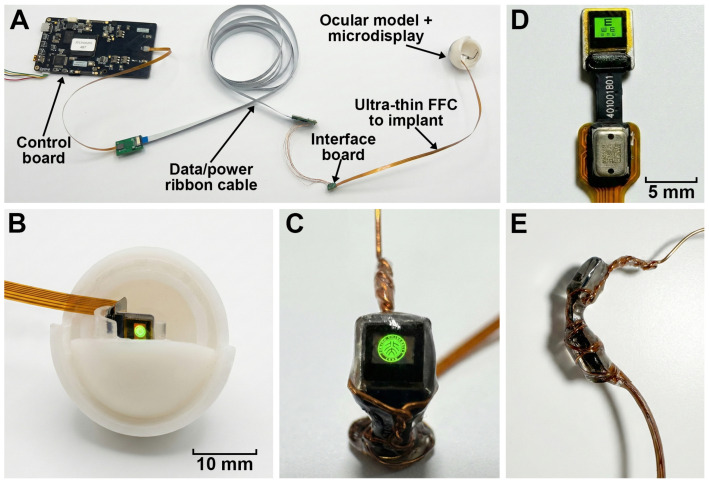
Implantable Micro-LED epiretinal display system and packaged implant. (**A**) Benchtop system including the control board, data/power ribbon cable, interface board, ultra-thin FFC to the implant, and a 3D-printed ocular model with an intraocular microdisplay. (**B**) Placement of the bare Micro-LED microdisplay at the posterior pole of the ocular model, illustrating the intended epiretinal location. (**C**) Close-up view of the bare microdisplay showing a high-contrast test pattern rendered on the 640×480-pixel array. (**D**) Front view of the unpackaged Micro-LED module and driver on the FFC, displaying a miniature eye-chart–like “E” pattern; based on the microdisplay geometry and retinal magnification, the pixel pitch imposes a Nyquist-limited sampling ceiling of approximately 20/17 under human eye scaling; this represents a geometric upper bound, not a prediction of functional behavioral acuity. (**E**) Side view of the PDMS-encapsulated implant with a copper-mesh arch, designed to follow the ocular curvature. Scale bars: 10 mm in (**B**); 5 mm in (**D**).

An overview of the complete benchtop system, including the control board, data/power ribbon cable, ultra-thin FFC, and the 3D-printed ocular model with the intraocular microdisplay, is shown in Figure 2A,B. A close-up view of the unpackaged Micro-LED module and its driver on the FFC, together with a high-contrast eye-chart–like test pattern rendered on the 640×480 array, is provided in Figure 2D.

### 2.2. Structural Design and Biocompatible Packaging of the Implantable Epiretinal Microdisplay

**Figure 3:**
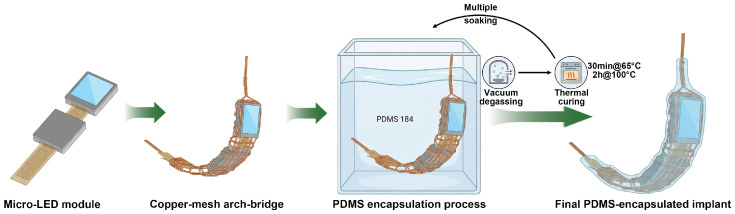
Schematic fabrication workflow of the metal–polymer composite epiretinal implant. A curved Micro-LED microdisplay with an attached FPC cable is first wrapped on its back and edges by a single 300μm annealed copper wire configured as a mesh arch-bridge to provide mechanical reinforcement and match the ocular curvature. The copper-wrapped assembly then undergoes multiple coating cycles, each consisting of immersion in Sylgard^®^ 184 PDMS, vacuum degassing at −90kPa for 30min, and a two-stage thermal cure (0.5h at 60 °C followed by 2h at 100 °C). Repeating this “immersion–degassing–curing” sequence yields a stable, uniform PDMS layer (<1 mm) and a flexible, biocompatible epiretinal implant in which the copper mesh and microdisplay remain visible through the transparent encapsulation.

The implant employs a metal–polymer composite arch-bridge structure (Figure 2C,E and Figure 3). As schematically illustrated in Figure 3, a 300μm-diameter annealed copper wire configured as a mesh is first wrapped around the back and edges of the curved Micro-LED microdisplay to provide mechanical reinforcement, approximate the ocular curvature, and facilitate reliable intraocular deployment. For encapsulation, the copper-wrapped assembly is then coated with Sylgard^®^ 184 PDMS (Dow Corning Corporation, Midland, MI, USA) to provide a biocompatible and mechanically compliant interface. Each coating cycle consists of immersion in uncured PDMS, vacuum degassing at −90kPa for 30min, and a two-stage thermal cure (0.5h at 60 °C followed by 2h at 100 °C), yielding a continuous, flexible coating with a thickness below 1mm, as verified by vernier caliper measurements. The resulting PDMS-encapsulated implant is shown in front and side views in Figure 2C,E, where both the copper mesh and the microdisplay remain clearly visible through the transparent encapsulation.

### 2.3. Bioengineering Design Rationale of the Epiretinal Implant

The mechanical architecture and encapsulation of the epiretinal implant were specifically tailored to reduce tissue–device mismatch and mitigate intraocular inflammation. The metal–polymer arch-bridge structure allows the Micro-LED chip to be supported by an annealed copper-wire mesh that can be gently bent to approximate the posterior globe curvature in canine eyes, while the surrounding PDMS substrate provides a compliant interface to the retina. By distributing mechanical loads over a larger contact area and avoiding sharp edges, this configuration is intended to decrease local stress concentrations and shear forces on the epiretinal surface, thereby reducing the risk of retinal microtrauma and chronic irritation.

Encapsulation thickness and material selection were also guided by bioengineering considerations. Sylgard^®^ 184 PDMS was chosen because of its reported ocular biocompatibility and its low elastic modulus, which allows the encapsulated implant to conform to the eye wall more readily than rigid coatings such as polyimide or silicon oxide. The PDMS layer is designed to be thin enough to limit added bulk while still providing a continuous barrier against fluid ingress and mechanical damage. In addition, the copper mesh embedded within the PDMS serves as both a mechanical backbone and a heat-spreading layer, helping to homogenize strain and dissipate localized stresses at the implant–tissue interface.

Finally, the electrical and thermal design of the system was optimized to balance optical performance with intraocular safety. The use of a high-efficiency Micro-LED microdisplay with total power consumption on the order of 30mW, combined with short duty cycles during stimulation, was intended to limit temperature rise at the retinal surface. The ultra-thin FFC reduces cable bulk and bending stiffness, while the copper mesh and surrounding ocular tissues provide additional pathways for heat dissipation. Although detailed thermal mapping was beyond the scope of this initial proof-of-concept study, these design choices were made to keep operating conditions within commonly accepted safety margins for intraocular devices and will be further quantified in future chronic experiments.

### 2.4. Finite-Element Bioheat Simulation of Intraocular Temperature Rise

Here, we briefly describe the finite-element bioheat model used to estimate intraocular temperature rise during continuous operation of the implant. Finite-element bioheat simulations were performed using COMSOL Multiphysics 6.3 (COMSOL AB, Stockholm, Sweden) with the Bioheat Transfer interface. We constructed a simplified two-dimensional axisymmetric model of the canine eye consisting of a vitreous body, a combined retina–choroid layer, and an outer scleral shell. The epiretinal implant was represented as a rectangular Micro-LED chip encapsulated in a thin PDMS layer and positioned along the inner surface of the posterior globe. Thermal properties (density, specific heat, and thermal conductivity) for ocular tissues and PDMS were taken from the literature [30,31,32,33,34]. The Pennes bioheat equation with perfusion was solved with a constant body-temperature boundary condition of 36.5 °C prescribed at the outer scleral surface. A uniform volumetric heat source corresponding to the worst-case electrical power of the microdisplay (30 mW) was applied to the active Micro-LED area. Transient simulations up to 300 s were performed, and the maximal temperature and temperature rise were extracted at the retinal surface and in neighboring intraocular tissues. Detailed ocular dimensions and material thermal properties used in the model are provided in Supplementary Tables S1–S3. All geometric parameters were drawn from published canine ocular biometry rather than direct measurements of the study animal, as individual biometry was not obtained; the simulation therefore provides a conservative first-pass engineering estimate rather than an animal-specific thermal prediction.

## 3. Surgical Procedures and Multimodal Evaluation

### 3.1. Animals and Ethical Approval

An adult male beagle dog (≈72 weeks old) was used in this study. All animal procedures were approved by the Institutional Animal Care and Use Committee of Peking University Health Science Center (PKUHSC, Approval No. PUIRB-LA2022650) and were conducted in accordance with institutional guidelines for animal welfare and ethical use.

### 3.2. Pre-Operation Preparations

One day before surgery, periocular hair was clipped to reduce contamination risk. Immediately prior to the procedure, the eyelids and surrounding periocular skin were scrubbed with sterile cotton swabs soaked in 10% povidone–iodine, and a drop of 5% povidone–iodine was instilled into the conjunctival sac for 2 min before irrigation with sterile saline. Xenogeneic porcine scleral graft was prepared two days in advance and stored at −40 °C. On the day of surgery, the graft was thawed, disinfected by immersion in povidone–iodine solution for 10 min, and then rinsed thoroughly with sterile saline prior to implantation.

### 3.3. Surgical Procedures

**Figure 4:**
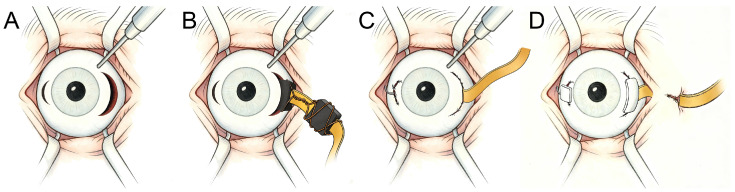
Schematic illustration of the surgical procedure (Transscleral Implantation Technique Covered by a Xenogeneic Scleral Patch). (**A**) Creation of scleral incisions at the 3 o’clock and 9 o’clock positions. (**B**) Insertion of the implant through the main incision at 3 o’clock. (**C**) Engagement and externalization of the integrated metallic anchoring end of the implant through the auxiliary incision at 9 o’clock, followed by closure of both scleral wounds. (**D**) Coverage of the externalized anchoring end and cable with xenogeneic scleral grafts, with the cable subsequently tunneled into the subcutaneous tissue.

The surgical method, termed TIT-XSP technique (Figure 4 and Supplementary Figure S1), was employed for device implantation. After conjunctival peritomy, a 25-gauge pars plana infusion cannula was placed at the 2 o’clock position to maintain intraocular pressure. At 3 o’clock, approximately 3mm posterior to the limbus, a 4mm scleral tunnel was created with a crescent blade, through which the integrated metallic anchoring end of the implant was inserted into the vitreous cavity. An auxiliary 2mm scleral incision was made at 9 o’clock, and a 20-gauge syringe was used to engage and externalize the integrated metallic anchoring end, enabling the implant to be centered along the visual axis. The external cable was routed through the main incision, which was closed with 8-0 absorbable sutures and reinforced with a 5×5mm scleral graft. The externalized anchoring end at 9 o’clock was fixed to the sclera and covered with a 3×3mm graft. The infusion cannula was then removed, and the conjunctiva was repositioned. The cable was tunneled subconjunctivally toward the lateral canthus, embedded within subcutaneous tissue through a 5cm skin incision, and secured with layered closure using 6-0 non-absorbable sutures. Finally, ofloxacin ointment was applied, and the ocular and skin incisions were covered with sterile gauze.

### 3.4. Post-Operation Care and Safety Evaluation

Following recovery from anesthesia, butorphanol was administered once daily for three days to provide postoperative analgesia. Levofloxacin ophthalmic solution and prednisolone acetate eye drops were applied to the operated eye three times daily for seven days. Starting on postoperative day 1, intravenous levofloxacin was administered once daily for three days to reduce systemic infection risk. Immediately after surgery, B-scan ultrasonography (Mindray Bio-Medical Electronics Co., Ltd., Shenzhen, Guangdong, China; Model: S7 SCI) was performed to verify implant positioning and retinal integrity. Ophthalmic examinations were conducted on postoperative days 1, 3, 5, and 7, including intraocular pressure assessment by digital palpation (reported as Tn or T-1; quantitative tonometry was not performed because concurrent corneal epithelial defects on days 1–3 contraindicated direct tonometric contact) and microscopic evaluation of ocular structures and inflammation. On postoperative day 7, anterior segment photography, wide-field color fundus photography, and swept-source optical coherence tomography were performed.

### 3.5. Visual Evoked Potential (VEP) Evaluation

Flash-evoked VEPs were recorded from a beagle (n=1) on postoperative days 3, 5, and 7 using an EBNEURO BE PLUS PRO STANDARD (EBNEURO S.r.l., Turin, Italy; Model: BE PLUS PRO STANDARD) (1024 Hz sampling, impedance <5 kΩ) to compare cortical responses between preoperative external white-LED flashes (85 lux, 1.05 Hz, 950 ms) and postoperative intraocular Micro-LED stimulation (85 lux, 125 lux, 175 lux; blank/grid/bar patterns; 1.052 Hz; 950 ms). The contralateral eye was occluded, and illuminance was calibrated via a DELIXI DLY-1802 (DELIXI Electric Co., Ltd., Yueqing, Zhejiang, China; Model: DLY-1802). Data from an 8-channel montage were analyzed primarily at Oz.

In MATLAB R2021b, continuous EEGs were segmented (−100 ms to 500 ms), baseline-corrected (−100 ms to 0 ms), and wavelet-filtered (4 Hz to 32 Hz). Artifacts >200 μV at Oz were rejected, retaining >100 artifact-free epochs per condition for ensemble averaging.

Waveform similarity (0 ms to 200 ms) was quantified via lag-tolerant normalized cross-correlation of z-scored signals. To evaluate within-session repeatability, N75/P100 latencies and peak-to-peak amplitudes were extracted from six overlapping moving-window sub-averages (80 trials/window, 20-trial step); a demonstration of VEP stability using this sliding window approach is provided in Supplementary Figure S3. Reported dispersions (mean ± SD) explicitly reflect within-session, trial-to-trial variability rather than independent biological replication.

### 3.6. Assessing Globe Integrity by Optical Coherence Tomography

Pupils were dilated with 1% tropicamide, and the eyes were manually aligned with the lens of a full-range swept-source OCT system (TowardPi Medical Technology Co., Ltd., Beijing, China; full-range swept-source OCT system) Bilateral anterior and posterior segment scans were acquired to evaluate structural changes of the globe.

### 3.7. Gross Photography

On postoperative day 7, the animal was humanely euthanized in accordance with institutional animal care guidelines, and both eyes were enucleated. Gross photography was performed to document the overall globe morphology and implant position.

## 4. Results

### 4.1. Ex Vivo Functional Validation of the Explanted Implant Device

Following euthanasia and explantation on postoperative day 7, the prototype was subjected to immediate in vitro functional testing to assess structural integrity and performance retention after physiological exposure. Visual and microscopic inspection showed no delamination, cracking, or leakage in the PDMS encapsulation or copper-mesh framework, and the FFC interfaces remained intact without fracture or moisture ingress. Upon reconnection to the original control board, the explanted microdisplay successfully displayed programmed sequences—including blank flashes, grid patterns, stripe patterns, and video streams—without image tearing or dead pixels. Core optoelectronic parameters, including illuminance (85±5.3lx) and power consumption (28.3±0.5mW), remained comparable to pre-implantation baselines.

### 4.2. Simulated Intraocular Temperature Rise During Continuous Operation

**Figure 5:**
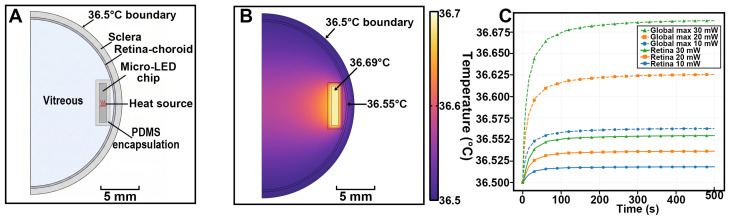
Thermal modeling of an intraocular Micro-LED epiretinal implant. (**A**) Axisymmetric 2D geometry of the eyeball used for bioheat simulations, showing vitreous, retina–choroid composite layer, sclera, and a PDMS-encapsulated Micro-LED chip positioned at the posterior pole. The outer scleral surface is constrained to a constant 36.5 °C to represent heat exchange with well-perfused orbital tissue, and the Micro-LED is modeled as a localized volumetric heat source. (**B**) Steady-state temperature distribution for a 30 mW operating power, illustrating the confined warm region around the implant and minimal temperature elevation at the retina–choroid interface. (**C**) Time course of global maximum temperature in the model and retinal temperature for driving powers of 10, 20, and 30 mW, showing rapid saturation within a few hundred seconds and a small absolute temperature increase (<0.2 °C at 30 mW), indicating a wide thermal safety margin for the proposed device.

Finite-element bioheat simulations were performed to estimate intraocular temperature rise during continuous operation of the epiretinal Micro-LED display at the worst-case electrical power of 30 mW (Figure 5). Using the simplified axisymmetric eye model shown in Figure 5A, a body-temperature boundary of 36.5 °C was imposed at the outer scleral surface, and a uniform heat source was applied to the Micro-LED chip embedded in the PDMS-encapsulated implant. The system approached a quasi–steady state after a hundred seconds of continuous operation, and the corresponding steady-state temperature distribution is illustrated in Figure 5B.

At steady state, the maximal temperature occurred near the implant–vitreous interface and reached approximately 36.69 °C, i.e., a local temperature rise of only ∼0.2 °C above the 36.5 °C boundary condition. The nearby retinal surface temperature was even lower (36.55 °C), corresponding to a retinal temperature increase of ∼0.05 °C relative to baseline. Temperatures elsewhere in the vitreous and along the posterior globe remained close to 36.5 °C, indicating that heat dissipation through the surrounding tissues effectively limited intraocular heating. These simulations provide a first-pass engineering estimate suggesting a conservative thermal safety margin under the modelled conditions; the predicted local temperature rise near the implant surface (<0.2 °C at 30 mW) and retinal surface temperature increase (∼0.05 °C) both remain far below the 2 °C limit of ISO 14708 [35]. Animal-specific biometry and direct intraocular thermometry would be required to confirm these estimates in individual subjects.

### 4.3. Visual Evoked Potentials Induced by Artificial Versus Natural Visual Stimulation

**Figure 6:**
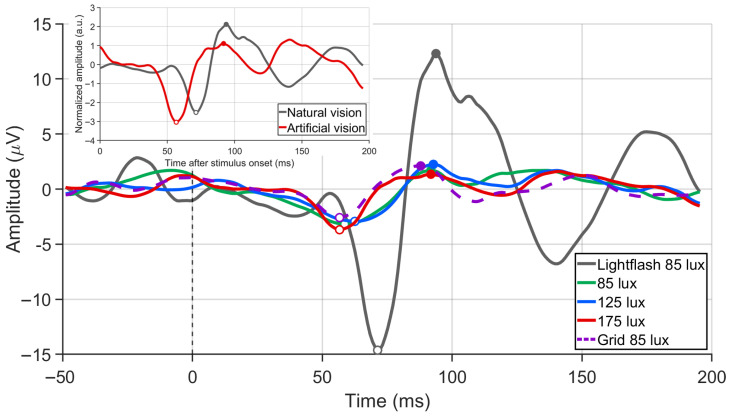
Flash-evoked transient VEPs in the implanted eye (*n* = 1). Main panel: Oz-channel grand averages (postoperative day 5) comparing preoperative white-LED flashes (85 lux, gray) with intraocular Micro-LED stimulation at 85 (green), 125 (blue), and 175 lux (red), alongside an 85 lux grid pattern (magenta dashed). Each trace averages >100 artifact-free epochs (rejection threshold: 200 μV). Inset: Zero-normalized waveforms for the white-LED (gray) and 175 lux Micro-LED (red) conditions. Waveform morphological similarity between the 175 lux Micro-LED and natural-vision grand-average waveforms was quantified by normalized cross-correlation (r=0.703 at a lag of 14.6 ms). Time 0 indicates stimulus onset.

After standardized preprocessing, the Oz-channel waveforms exhibited a discernible N1–P1 complex under both preoperative external white-LED flash stimulation and postoperative intraocular Micro-LED stimulation (Figure 6), indicating broadly similar transient temporal dynamics. The z-score-normalized waveforms at 175 lux (inset of Figure 6) further illustrated the morphological similarity between conditions, with a normalized cross-correlation peak of r=0.703 at a temporal lag of 14.6 ms.

Qualitative assessment of the sliding-window sub-averages demonstrated within-session waveform consistency, with individual window waveforms broadly aligning with the overall grand average. As illustrated in Supplementary Figure S3, modest variations were observed between different time windows within a session; these reflect within-session trial-to-trial variability and should not be interpreted as cross-session or cross-subject reproducibility given the single-animal design (n=1).

Across the tested Micro-LED intensities, component latencies showed no clear systematic shift with increasing illuminance. At 175 lux, the N1 latency was 56.84±0.44 ms under intraocular Micro-LED stimulation, compared with 71.13±0.40 ms under external white-LED flashes; the P1 latency was 91.21±0.53 ms versus 96.03±6.10 ms, respectively (mean ± SD across sliding-windows sub-averages).

In contrast, peak-to-peak VEP amplitudes elicited by intraocular Micro-LED stimulation were substantially attenuated relative to natural vision. Amplitudes at 85, 125, and 175 lux were (6.88±1.23) μV, (5.53±1.30) μV, and (6.11±0.67) μV, respectively, compared with (31.28±15.27) μV for external white-LED flashes. The artificial responses showed minimal intensity-dependent amplitude changes, with the overall range remaining notably compressed compared to natural stimulation. Notably, all metrics were derived from sliding-window sub-averages within a single recording session (n=1 animal); the reported standard deviations therefore reflect within-session temporal variability rather than inter-subject biological variance.

### 4.4. Postoperative Regular Ophthalmic Examinations

**Figure 7:**
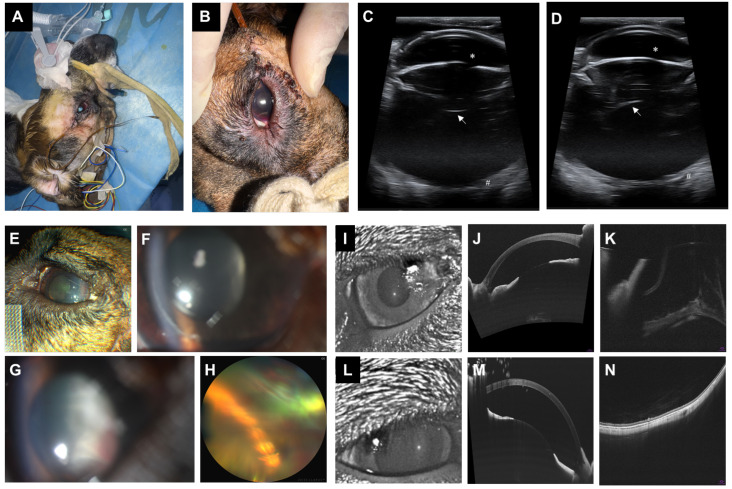
Postoperative gross and ophthalmic evaluations. (**A**,**B**) Gross appearance of the canine head and operated eye immediately after implantation. (**C**,**D**) B-scan ultrasonography immediately after surgery, showing the iris and pupil plane (∗), attached retina (#), and confirmation of implant position at the lens plane (arrows). (**E**–**H**) Ophthalmic examinations at 1 week postoperatively: (**E**) external view of the eye; (**F**) anterior segment image showing mild hyphema in the inferior chamber; (**G**) anterior segment image showing a membranous structure posterior to the lens; (**H**) fundus photograph showing the implant inferiorly with findings superiorly consistent with, but not definitively confirming, retinal detachment; (**I**–**N**) OCT imaging, with (**I**–**K**) left eye (implanted) and (**L**–**N**) right eye (control). (**I**,**L**) Infrared images; (**J**,**M**) anterior segment OCT showing comparable anterior chamber depth in both eyes; (**K**,**N**) fundus OCT showing poor visualization in the left eye with findings consistent with retinal detachment (subsequently confirmed on gross examination after euthanasia), while the right retina remained attached.

Immediately after surgery, a small hemorrhage was observed on the iris surface, and the implant was located posterior to the lens (Figure 7A,B). No wound leakage was detected, and intraocular pressure (IOP) was within the normal range (Tn). On postoperative day 1, IOP was slightly reduced (T-1). Mild corneal epithelial defects and stromal edema were noted, accompanied by a small hyphema in the inferior anterior chamber. The implant remained poorly visible. On postoperative day 3, IOP remained mildly reduced (T-1). Corneal edema persisted, with residual hyphema still present, and the implant posterior to the lens was obscured. By postoperative days 5 and 7, IOP had returned to normal (Tn), and corneal edema showed signs of resolution. A faint fibrin-like proliferation was vaguely visible posterior to the lens.

### 4.5. Postoperative Ophthalmic Imaging

Immediate postoperative B-scan ultrasonography showed no gross abnormalities in the anterior segment and revealed a faint reflective signal posterior to the lens, consistent with the implanted device (Figure 7C,D). Importantly, no abnormal echoes were observed in the retinal plane, providing no initial sonographic evidence of retinal detachment and supporting correct acute positioning. At 1 week postoperatively, anterior-segment OCT demonstrated deep, well-formed anterior chambers in both eyes, without overt anterior-segment hemorrhage or severe inflammatory signs (Figure 7I–N). However, posterior-segment visualization in the operated eye was limited by residual media opacity; fundus photography and OCT showed findings consistent with, but not definitively confirming, retinal detachment (Figure 7H,K). Definitive confirmation of inferior retinal detachment was obtained on gross examination of the enucleated globe after euthanasia on day 7. The contralateral control eye remained attached on imaging (Figure 7N).

### 4.6. Gross Photography Results

Gross examination showed that the enucleated globes were normal in size (Supplementary Figure S2A). Bilaterally, the scleral grafts were well integrated with the surrounding tissues, showing no perforation or leakage (Supplementary Figure S2B,C). The crystalline lenses remained transparent without apparent damage (Supplementary Figure S2E,F). Crucially, at the 7-day postoperative time point, inspection of the explanted eyes revealed no gross evidence of significant tissue proliferation, overt inflammation, or fibrotic encapsulation around the intraocular device (Supplementary Figure S2D). However, despite this lack of acute foreign body response, mechanical instability was evident. Following cornea and lens removal, direct evaluation of the vitreous cavity confirmed downward rotation of the implant (Supplementary Figure S2G) and localized torsion of the external cable. Inferior retinal detachment in the operated left eye was confirmed on gross inspection of the enucleated globe; this finding had been suspected but not definitively established on postoperative ophthalmic imaging.

## 5. Discussion

The principal strengths of the present work are: (1) it provides the first in vivo demonstration of intraocular Micro-LED projection in a large-animal (canine) model, a scale substantially more relevant to human surgical translation than rodent eyes; (2) it employs a high-pixel-density commercial platform (640 × 480, 4 µm pitch) rather than a low-resolution custom prototype, establishing a realistic optoelectronic baseline; (3) it integrates device, encapsulation, flexible cabling, and surgical approach in a single end-to-end implantation; and (4) it reports adverse events—device rotation and retinal detachment—transparently, providing failure-mode data that directly inform next-iteration design. These contributions are discussed in the context of remaining limitations below.

### 5.1. Encapsulation Performance and Thermal Safety

This study presents a pilot in vivo implantation of a VGA-resolution Micro-LED epiretinal microdisplay, based on a full-color–capable platform, in a canine eye, primarily as an acute probe of encapsulation performance and thermal safety over a 7-day observation window. Preservation of luminance, refresh rate, and power consumption, together with intact pixel function, supports the short-term robustness of the PDMS–copper mesh encapsulation against intraocular mechanical loading and fluid ingress.

With respect to longer-term performance, prior work on PDMS-based retinal prosthesis structures has reported accelerated-aging evidence consistent with multi-year encapsulation lifetimes (e.g., ≥2 years) in a foldable epiretinal device incorporating PDMS components [36]. In addition, PDMS-based materials have been explored in ocular implantation contexts with generally acceptable short-term tissue compatibility in rabbits [37]; however, long-term retinal outcomes can depend strongly on surface chemistry and device–tissue interactions, as suggested by reports where modified PDMS scaffolds outperformed unmodified PDMS in extended subretinal follow-up [38]. More broadly, chronic implants can elicit foreign-body responses (e.g., fibrotic encapsulation or gliosis) even when encapsulation remains intact, and softer, more compliant materials are often pursued to mitigate mechanical mismatch and tissue response [39].

Importantly, polymer–metal interfaces are recognized failure-prone pathways for moisture ingress and delamination in chronic implant packaging; for example, accelerated-aging failure driven by water penetration through polymer–metal interfaces has been documented in an all-polymer retinal prosthesis platform [40]. Accordingly, future work will focus on engineering the PDMS–mesh interface (e.g., surface activation and silane-based coupling chemistries, or adhesive interlayers) to improve interfacial adhesion and reduce delamination risk [41]. In parallel, finite-element bioheat simulations under worst-case continuous operation (30 mW) predicted only a minimal retinal temperature increase (<0.1 °C), which is far below the 2 °C temperature-rise criterion commonly used in ISO 14708 for implantable devices [35]. Overall, the present results establish short-term in vivo reliability of the encapsulation and motivate dedicated accelerated-aging tests and chronic in vivo studies to quantify long-term stability and tissue response. Regarding the mechanism of intraocular temperature rise: the simulated elevation arises from Joule heating within the Micro-LED chip—electrical power not converted to photons is dissipated as heat, which accumulates locally because the vitreous has low thermal conductivity (∼0.594 W m^−1^ K^−1^) before being conducted to the perfused scleral boundary. No direct intraocular temperature measurement was performed in the present study; all thermal data are model-derived, a limitation acknowledged in Section 2.4.

### 5.2. Photometric Dose and Photobiological Safety

To better contextualize the optical dose delivered to the retina under natural versus artificial stimulation, we performed a simple photometric–radiometric conversion using the measured illuminances. In our VEP protocol, the natural flash condition was adjusted to a mean illuminance of 85 lux at the eye, whereas the brightest artificial condition used a mean illuminance of 175 lux. For monochromatic green light around 525 nm, 1 lux corresponds to approximately 1/683 W/m^2^ of irradiance under photopic conditions [42]. Thus, the natural flash yields an estimated retinal irradiance of $E_{\mathrm{nat}}\approx 85/683\approx 0.12$ W/m^2^, while the brightest artificial condition yields $E_{\mathrm{art}}\approx 175/683\approx 0.26$ W/m^2^. Even under a conservative assumption of 300 s of continuous exposure, these correspond to radiant exposures of $H_{\mathrm{nat}}\approx 0.12\times 300\approx 0.004$ J/cm^2^ and $H_{\mathrm{art}}\approx 0.26\times 300\approx 0.008$ J/cm^2^, respectively. In the actual VEP recordings, the duty cycle of both stimuli was substantially lower than 100%, so the effective radiant exposures were smaller still. Both calculated irradiance values are well below the ISO 15004-2 retinal photochemical limit (2.2 W/m^2^) and are four orders of magnitude lower than the thermal retinal limit (7000 W/m^2^) [43].

### 5.3. Advantages over Electrical and Prior Optical Retinal Prostheses

Our Micro-LED optical-projection paradigm represents a substantive shift from electrode-based retinal prostheses by replacing direct electrical contact and mechanical tacking with physiological photostimulation. Electrode systems such as the Argus II require chronic epiretinal contact and mechanical fixation; in post-approval series, approximately 26–30% of patients experienced at least one serious adverse event, and system costs exceeding USD 100,000 further contribute to procedure-related morbidity and limited accessibility [9,17,44]. By contrast, an optically driven Micro-LED display delivers patterned light to intact photoreceptors and retinal circuitry without physical tacks or charge injection, theoretically reducing tack-related mechanical injury and stimulation-related cytotoxicity while preserving the retina’s intrinsic signal processing.

Beyond these advantages over electrical implants, the present prototype also addresses key limitations of earlier optical projection-based systems. Prior intraocular projectors were typically bulky, monochromatic, and of limited brightness, constraining their feasibility for long-term implantation [27]. In comparison, our device integrates a compact 0.13-inch Micro-LED microdisplay (16.8×5.1×1.78 mm) with VGA resolution (640×480 pixels) and high peak luminance (up to $4000\;\mathrm{cd}/m^{2}$). Although the prototype tested in vivo employed a green-emitting module at 525 nm, identical devices are available in full-color variants [45], underscoring a clear path toward full-color artificial vision. In addition, the module supports a simple, high-speed digital interface (Quad-SPI, up to 128 Mbit/s), facilitating seamless control and real-time image delivery. Together, these attributes confer significant advantages in miniaturization, resolution, brightness, and scalability compared with earlier intraocular projection platforms, and our short-term in vivo safety and VEP data provide preliminary empirical support for this concept.

### 5.4. Visual-Performance Considerations: Field of View and Theoretical Resolution

From a visual-performance standpoint, the present geometry imposes constraints on the achievable field of view and sampling-limited resolution. Assuming near-unity magnification between the Micro-LED array and the retinal surface in a human-sized eye, the 2.64×2.02mm emitting area would subtend approximately $9^{\circ }\times 7^{\circ }$ of the central visual field (using a retinal magnification factor of ∼0.29 mm/deg). With a 4μm pixel pitch, this corresponds to ∼70 pixels/deg and a Nyquist-limited spatial frequency of ∼36 cycles/deg, representing a pixel-pitch-constrained Nyquist sampling ceiling of approximately 20/17 Snellen (logMAR ≈−0.08). This is a geometric upper bound imposed by display sampling under ideal optical conditions; actual functional acuity would require behavioral grating-detection assays and is expected to be substantially lower. In the canine eye used here, these physical dimensions translate to a somewhat larger central field but lower pixels/deg, still providing a sampling density that, at least at the sampling limit, exceeds that associated with the best-reported visual acuities of current electrode-based retinal prostheses (approximately 20/460) [12] and earlier Micro-LED intraocular projection prototypes (approximately 20/130) [27].

### 5.5. Cortical Responses to Artificial Vision

In the present experiment, artificially evoked VEP waveforms exhibited a clear N75–P100 complex that closely resembled responses elicited by natural vision, and were also consistent with waveforms previously reported under electrical stimulation paradigms [46], further supporting the physiological relevance of the response. Latencies under artificial stimulation showed no significant differences compared with natural vision, indicating preserved temporal characteristics of early visual processing. By contrast, VEP amplitudes under artificial stimulation were markedly smaller than under natural vision, and the pattern was non-monotonic across the three illuminance levels tested. Several factors likely contribute to this attenuation. First, the green prototype (525 nm) falls outside the peak sensitivity of canine photoreceptors, reducing the effective photon catch relative to broadband white illumination [47]. Second, the 2.64×2.02 mm emitting aperture subtends a narrow central field, activating a substantially smaller retinal region than the full-field white-LED flash. Third, the finite device–retina separation further attenuates effective retinal irradiance. Fourth, implant rotation confirmed by postoperative day 7 suggests that incremental device displacement across recording sessions (days 3, 5, and 7) may have progressively shifted the stimulated retinal area, disrupting any monotonic dose–response relationship. The non-monotonic amplitude pattern is therefore at least partially consistent with progressive mechanical misalignment; whether a clearer intensity–response relationship would emerge with a stably positioned device remains to be established in future controlled experiments. Together, these findings suggest that the implant can effectively deliver optical stimulation to the upstream visual pathway and that the early visual cortex remains responsive to such input. At the same time, VEPs capture only neurophysiological responses and do not directly quantify functional vision or perceptual quality, underscoring the need for future behavioral studies to establish how closely Micro-LED–mediated vision approximates natural sight.

### 5.6. Postoperative Outcomes and Biomechanical Limitations

Short-term postoperative observations revealed successful transscleral implantation with no wound leakage and generally stable IOP. A mild iris hemorrhage was noted, which is common after such procedures and did not affect ocular function [44,48]. However, during the first 1–3 postoperative days, transient hypotony, corneal edema, and hyphema were observed, consistent with early postoperative findings reported after pars plana and transscleral procedures [49]. The transient IOP reduction (T-1 by digital palpation) most likely reflects a combination of: aqueous humor egress through the incompletely healed scleral tunnel; transient ciliary body hypofunction following pars plana cannulation; and inflammation-mediated suppression of aqueous secretion. IOP normalised to Tn by days 5–7, consistent with wound sealing and resolution of the acute inflammatory response. These changes were associated with temporary media opacities that reduced posterior visualization. Because sustained hypotony is a known risk factor for ciliochoroidal/choroidal detachment after intraocular surgery, future designs should prioritize less invasive implantation techniques and improved control of postoperative inflammation [49,50]. By days 5–7, IOP had normalized and corneal edema had resolved, although faint fibrinous proliferation behind the lens suggested early capsular reactions. Posterior segment visualization was limited by transient media opacities; imaging at day 7 suggested possible retinal detachment in the operated eye, which was subsequently confirmed on gross examination. While this 7-day macroscopic evaluation revealed no gross tissue proliferation or inflammation directly around the implant, extended histopathological assessments (e.g., at 30 days and 6 months) remain necessary to fully evaluate potential chronic foreign body reactions (FBR), gliotic responses, and long-term device integration, as fibrotic and/or gliotic responses have been reported at the retina–implant interface in chronically implanted retinal prostheses [51,52]. Notably, device rotation and torsion of the external cable were also observed, highlighting the need for design modifications—such as lighter intraocular components or additional scleral support structures—to improve stability and long-term performance.

From a biomechanical standpoint, the present arch-bridge design was sufficient to survive acute implantation but clearly suboptimal for long-term intraocular stability. By day 7, we observed a downward rotation of the device within the vitreous cavity and an associated inferior retinal detachment in the operated eye, together with torsion of the external cable along the scleral surface. These findings suggest that a single transscleral anchoring point at the 9 o’clock meridian cannot adequately counteract the gravitational torque and shear forces generated by eye movements and extraocular muscle action on a relatively heavy, eccentrically supported implant. Moreover, the current copper-mesh backbone and straight FFC exit likely concentrate mechanical load at the anchor and at the inferior retinal contact zone, predisposing to chronic traction.

Future iterations will therefore prioritize biomechanical optimization in parallel with optoelectronic performance. Strategies under consideration include reducing device mass and moment of inertia by thinning or partially replacing the copper mesh with lighter polymeric reinforcements [53], redesigning the support frame to shift the center of gravity closer to the scleral wall, and implementing multi-point fixation (for example, a circumferential scleral flange or two–to–three distributed anchors) to distribute load and minimize local traction on the retina [54]. In addition, routing the cable through a shorter intrascleral tunnel with strain-relief loops, rather than a long subconjunctival track, may decrease torsional stress at the anchor [55]. These design changes, ideally guided by subject-specific finite-element models of ocular biomechanics under saccades and head movements [54], will be essential to transform the present acute engineering-validation prototype into a chronically stable epiretinal interface suitable for clinical translation.

### 5.7. Integration with Optogenetic and Hybrid Electro-Optical Strategies

Combining Micro-LED stimulation with contemporary optogenetic strategies could further expand clinical impact. Recent clinical trials of optogenetic gene therapy have shown that rendering inner retinal neurons light-sensitive can partially restore visual function in advanced photoreceptor loss when paired with engineered light-delivery systems—specialized devices that deliver spectrally, spatially, and temporally controlled light [29,56,57,58]. An implantable, high-resolution Micro-LED array could provide the precise, patterned illumination needed to activate optogenetically sensitized neurons at physiologically relevant scales, while inherently adapting to rapid eye movements due to its intraocular placement. This approach may improve sensitivity, spatial resolution, and perceptual richness compared with bulky external goggles or broad-field illumination, offering a promising route to extend optical artificial-vision strategies to patients with photoreceptor degeneration and to broaden their clinical applicability. In line with this, Roh et al. demonstrated that combining optogenetic stimulation with low-amplitude electrical pulses significantly enhanced retinal ganglion cell responses while reducing the required optical power [59]. Such hybrid electro-optical paradigms may mitigate the high-intensity light demands and phototoxicity risks that currently limit purely optogenetic approaches, further underscoring the potential of Micro-LED–based implants as versatile platforms for future artificial vision.

### 5.8. Study Limitations and Future Directions

Despite these encouraging short-term results, several limitations remain and should temper interpretation of the present data. From an engineering standpoint, the current device and TIT-XSP procedure should still be regarded as a single-animal, acute biomechanics and safety probe rather than a chronically optimized implant. The 7-day observation window is insufficient to predict chronic fibrotic encapsulation, delayed inflammatory reactions, immune-mediated responses, or long-term mechanical stability at the implant–tissue interface; extended implantation studies with serial imaging and histopathology will be required to establish true biocompatibility and durability.

In addition, the present surgical workflow relied on a relatively large, non-foldable epiretinal module and sizeable scleral tunnels, which likely contributed to the early hypotony and transient anterior segment inflammation observed postoperatively; translation toward foldable or extensible formats and smaller, self-sealing incisions will be essential to reduce tissue trauma and simplify implantation. Moreover, both the finite-element bioheat model and optical safety estimates were intentionally simplified and conservative; direct intraocular thermometry and longitudinal structural imaging will be needed to confirm the predicted wide thermal and photobiological safety margins during chronic operation.

Although the canine model approximates human ocular anatomy more closely than rodent eyes, species differences in globe size, scleral thickness, ocular biomechanics, and postoperative behavior may alter implant kinematics and wound healing, so extrapolation to human patients must remain cautious. Finally, while VEP recordings provided preliminary evidence of primary visual cortex activation, they provide only a neurophysiological surrogate for vision and do not address perceptual quality or functional benefit; future work should incorporate chronic implantation and behavioral assays—such as pattern discrimination, motion perception, and navigation tasks—to determine how effectively Micro-LED–mediated intraocular projection can restore useful vision and to guide subsequent human translation.

## 6. Conclusions

In this study, we developed a high–pixel-density, full-color–capable Micro-LED epiretinal microdisplay designed to bypass anterior segment opacities by projecting patterned light onto intact retinal photoreceptors, and we performed a pilot in vivo evaluation in a single beagle with 7 days of follow-up (*n* = 1). The TIT-XSP transscleral approach enabled intraocular placement, and copper-mesh–reinforced PDMS encapsulation preserved device integrity during the observation window. Finite-element bioheat simulations and photometric–radiometric analysis suggested minimal retinal temperature rise and optical exposure within relevant safety limits under the modeled assumptions. Micro-LED stimulation elicited flash-evoked VEPs with consistent within-session waveform morphology (normalized cross-correlation r=0.703 at 14.6 ms lag versus natural-vision grand average), providing preliminary neurophysiological surrogate evidence of early visual pathway responsiveness; given the single-animal design (n=1), these findings should not be interpreted as cross-subject replication. Short-term follow-up nonetheless revealed hypotony, inflammation, implant rotation, and inferior retinal detachment, emphasizing the need for biomechanical optimization, chronic implantation studies, and multi-animal replication with behavioral endpoints. Overall, this work establishes an intraocular Micro-LED optical interface platform and defines key engineering and validation requirements that must be addressed before longer-term functional claims can be made.

## Data Availability

The original contributions presented in the study are included in the article. Further inquiries can be directed to the corresponding authors.

## Supplementary Materials

The following supporting information can be downloaded at: https://www.mdpi.com/article/10.3390/bioengineering13040397/s1, Figure S1: Surgical procedure for implantation; Figure S2: Gross examination of explanted eyes; Figure S3: VEP stability demonstration with sliding windows; Table S1: Key canine ocular geometry parameters relevant to vitreous-cavity implantation.; Table S2: Parameters of ocular tissues used for acoustic and thermal simulation; Table S3: Key parameters used in the COMSOL ocular thermal simulation model.
