# Supplementary material for: Intraocular Micro-LED Epiretinal Projection for Anterior Segment Blindness: Design and Large-Animal Feasibility Study
Source: Bioengineering (Basel). 2026 Mar 29;13(4):397. doi: 10.3390/bioengineering13040397 (PMC13113396; doi:10.3390/bioengineering13040397)
Supplement: Supplementary file 1 [file bioengineering-13-00397-s001.zip › bioengineering-4199257-supplementary.pdf]

Supplementary Material

Supplementary Tables

Table S1. Key canine ocular geometry parameters relevant to vitreous-cavity implantation.

Parameter	Symbol	Unit	Reference value	Notes (implant relevance)	Reference
Axial length of the globe	AXL	mm	20.9	Overall globe scale; bounds posterior-segment geometry.	[1]
Vitreous chamber anteroposterior distance	VCD	mm	9.6	Proxy for usable vitreous depth; affects clearance and device–retina separation.	[1]
Vitreous humor volume	V_vit	mL	1.70 ± 0.86	Cavity scale estimate; useful for thermal/power-density intuition.	[2]
Scleral shell radius of curvature	R_scl	mm	11.3 ± 0.4	Guides curvature matching and bending-radius requirements for conformal packaging.	[3]
Minimal equatorial scleral thickness	t_scl,equ	mm	0.34 ± 0.13	Relevant to transscleral entry, anchoring/suturing, and tear risk near the equator.	[2]

Regional scleral thickness (anterior / equatorial / posterior)	—	mm	0.58 ± 0.08 / 0.47 ± 0.05 / 0.68 ± 0.11	Location-specific thickness estimates for fixation/exit-site planning.	[3]

Notes: Values are reference measurements from normal canine eyes and may vary with breed, age, and measurement modality. AXL and VCD are from CT biometry (44 eyes). Vitreous volume and minimal equatorial scleral thickness are reported as mean ± SD. Scleral shell curvature and regional scleral

thickness are from B-mode ultrasound measurements (mean ± SD).

References:

1. Salgüero R, Johnson V, Williams D, Hartley C, Holmes M, Dennis R, Herrtage M. CT dimensions, volumes and densities of normal canine eyes. Vet Rec. 2015;176(15):386. doi:10.1136/vr.102940.

2. Gilger BC, Reeves K-A, Salmon JH. Ocular parameters related to drug delivery in the canine and equine eye: aqueous and vitreous humor volume and scleral surface area and thickness. Vet Ophthalmol. 2005;8(4):265-269. doi:10.1111/j.1463-5224.2005.00401.x.

3. Palko JR, Pan X, Liu J. Dynamic testing of regional viscoelastic behavior of canine sclera. Exp Eye Res. 2011;93(6):825-832. doi:10.1016/j.exer.2011.09.018.

Table S2. Parameters of ocular tissues used for acoustic and thermal simulation .

Tissues	Density (kg/m3)	Sound speed (m/s)	Heat capacity at constant pressure (J/kg/K)	Thermal conductivity (W/m/K)	Attenuation (dB/cm/MHz)
Water	1000	1500	4178	0.62	0
Cornea	1062	1586	4178	0.58	0.78
Aqueous	1007	1497	3997	0.59	0.01
Vitreous	1005	1532	3999	0.60	0.012
Lens	1076	1647	3000	0.40	1.19
Iris*	1090	1588	3421	0.49	0.62
Sclera	1088	1647	4178	0.58	0.97
Retina	1034	1538	3680	0.57	1.15

* Direct measurement results for iris were not available. Muscle parameters were used for the iris because it is mainly composed of muscle.2

References:

4. Nabili, M., Geist, C., and Zderic, V. (2015). Thermal safety of ultrasound-enhanced ocular drug delivery: A modeling study. Medical Physics, 42(10), 5604–5615.

5. Asano, N., Schlötzer-Schrehardt, U., and Naumann, G.O. (1995). A histopathologic study of iris changes in pseudoexfoliation syndrome. Ophthalmology, 102(9), 1279–1290.

Supplementary Methods

Supplementary Methods: Thermal Simulation Settings (COMSOL)

Thermal simulations were performed in COMSOL Multiphysics using a physics-controlled mesh (element size: Normal). The initial intraocular temperature was set to the physiological baseline (Tbody = 36.5 °C). The Micro-LED package was modeled as a uniform volumetric heat source with total electrical power P_led distributed within the device volume. Key model parameters are summarized in Table S3.

Table S3 Key parameters used in the COMSOL ocular thermal simulation model

Category	Parameter	Symbol	Value	Unit	Notes
Numerical settings	Mesh type	—	Physics-controlled	—	Element size: Normal
Numerical settings	Baseline temperature	Tbody	36.5	°C	Initial intraocular temperature
Geometry	Eye radius	R_eye	12	mm	Spherical eye approximation
Geometry	Sclera thickness	t_sclera	0.8	mm	
Geometry	Retina+choroid thickness	t_retina	0.25	mm	
Geometry	Vitreous thickness	t_vitreous	8	mm	
Geometry	Micro-LED width (on retinal surface)	led_w	5.12	mm	
Geometry	Micro-LED thickness	led_t	0.93	mm	
Geometry	Device–retina radial gap	gap_led	1	mm	Radial separation between LED and inner retinal surface
Geometry	PDMS encapsulation thickness	t_pdms	0.45	mm	

Heat source	Micro-LED electrical power	P_led	30	mW	Maximum typical operating power (datasheet bound); modeled as continuous load
Heat source	Equivalent volumetric heat generation	Q_led	1.057×105	W/m³	Applied uniformly within Micro-LED volume
Material properties	Vitreous density	ρ_vitreous	1005	kg/m³	

Material properties	Vitreous heat capacity	C_p,vitreous	3999	J/(kg·K)	
Material properties	Vitreous thermal conductivity	k_vitreous	0.60	W/(m·K)	
Material properties	Sclera density	ρ_sclera	1088	kg/m³	
Material properties	Sclera heat capacity	C_p,sclera	4178	J/(kg·K)	
Material properties	Sclera thermal conductivity	k_sclera	0.58	W/(m·K)	
Material properties	Retina density	ρ_retina	1034	kg/m³	
Material properties	Retina heat capacity	C_p,retina	3680	J/(kg·K)	
Material properties	Retina thermal conductivity	k_retina	0.57	W/(m·K)	
Material properties	PDMS density	ρ_pdms	970	kg/m³	
Material properties	PDMS heat capacity	C_p,pdms	1460	J/(kg·K)	
Material properties	PDMS thermal conductivity	k_pdms	0.16	W/(m·K)	
Perfusion	Retina perfusion rate	w_retina	0.02	$\frac{1}{s}$	Pennes bioheat perfusion term
Perfusion	Sclera perfusion rate	w_sclera	0.005	$\frac{1}{s}$	Pennes bioheat perfusion term

Note: R_led was defined as the radial location of the Micro-LED package center within the spherical eye geometry (R_led = R_eye _ t_sclera _ t_retina _ gap_led _ led_t/2). The volumetric heat source was

computed as Q_led = P_led / (2π R_led · led_w · led_t) and applied uniformly within the Micro-LED volume.

Supplementary Figure Legends

Supplementary Figure Legends (Surgical Procedure and Gross Examination)

Figure S1. Surgical procedure for implantation.

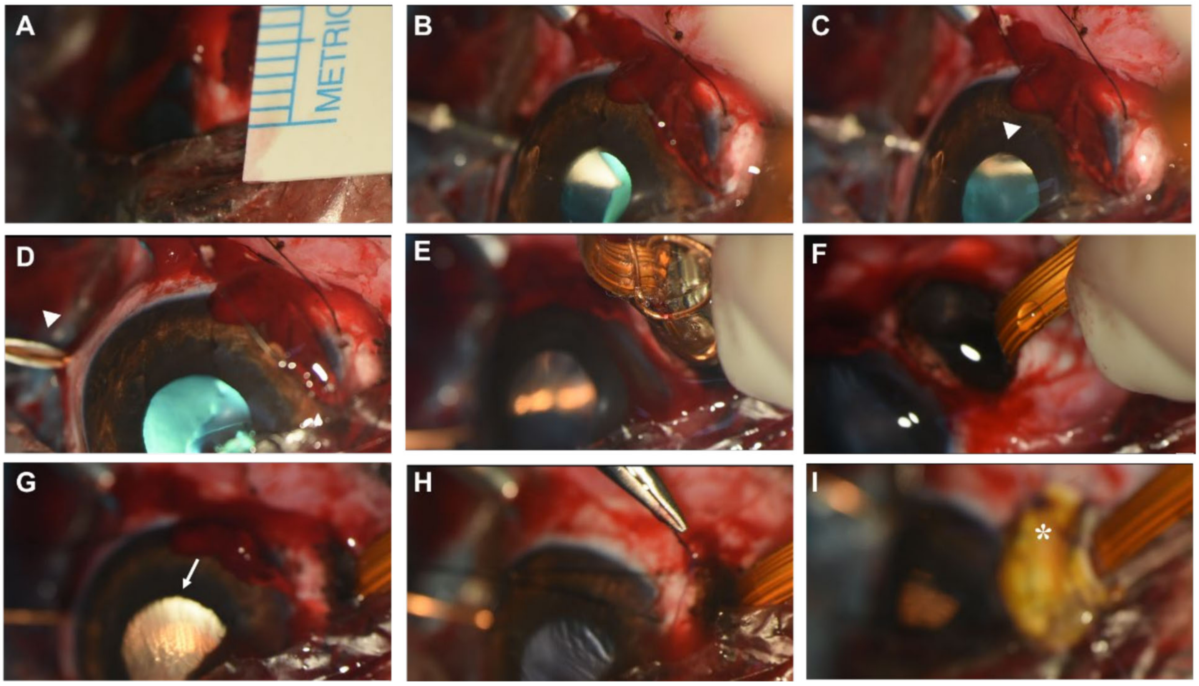

This figure documents the key steps in the surgical procedure for implanting the Micro-LED device using the transscleral implantation technique. Panels include: (A) creation of the main scleral incision at the 3 o'clock position; (B) insertion of a 20-mL syringe needle through the sclera at the 9 o'clock position; (C) introduction of the metallic anchoring device through the main incision at 3 o'clock and guidance into the syringe needle under direct visualization (arrow); (D) externalization of the anchoring device through the 9 o'clock incision by withdrawal of the syringe needle (arrow indicates the anchor within the needle); (E) insertion of the optical module through the main incision at 3 o'clock; (F) complete intraocular placement ofthe implant; (G) adjustment of the optical module to align with the pupillary center; (H) closure of the main incision with fixation of the external cable at the incision site; (I) coverage of the external cable with xenogeneic sclera (asterisk).

Figure S2. Gross examination of explanted eyes.

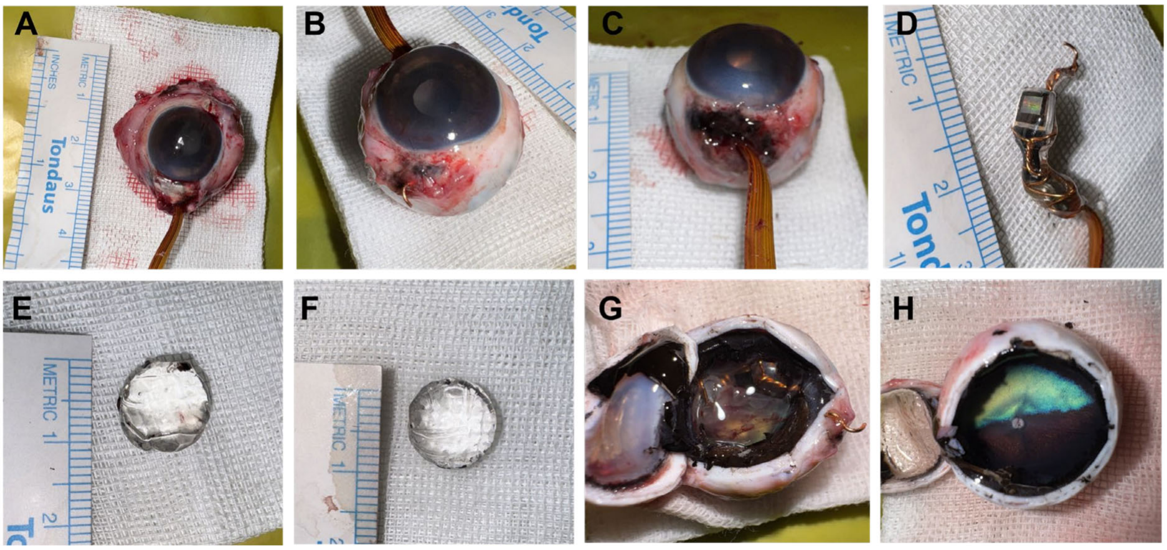

This figure provides the gross examination of explanted eyes. Panels include: (A) gross appearance after enucleation, showing normal globe size with bilateral xenogeneic scleral grafts well integrated into surrounding tissues without perforation or leakage; (B-C) after removal of the grafts, showing the spatial relationship between the implant and the globe, with localized torsion of the external cable; (D) Gross view of the explanted device, demonstrating the absence of surrounding tissue proliferation or fibrotic encapsulation. (E–F) comparison of crystalline lenses: (E) left (operated) eye and (F) right eye, both showing comparable transparency without apparent lens damage; (G) gross view of the left retina and implant, showing downward rotation ofthe implant with associated inferior retinal detachment; (H) gross view of the right retina, which remained attached.

Supplementary Figure Legends (VEP Stability Demonstration with Sliding Windows)

The following figure is included in the Supplementary Material to provide a demonstration of the stability of visual evoked potentials (VEPs) over time. Sliding window analysis was performed to evaluate VEP stability across different time windows. The corresponding figure legend is listed below, with a placeholder for the image (Figure 3).

Figure S3. VEP stability demonstration with sliding windows.

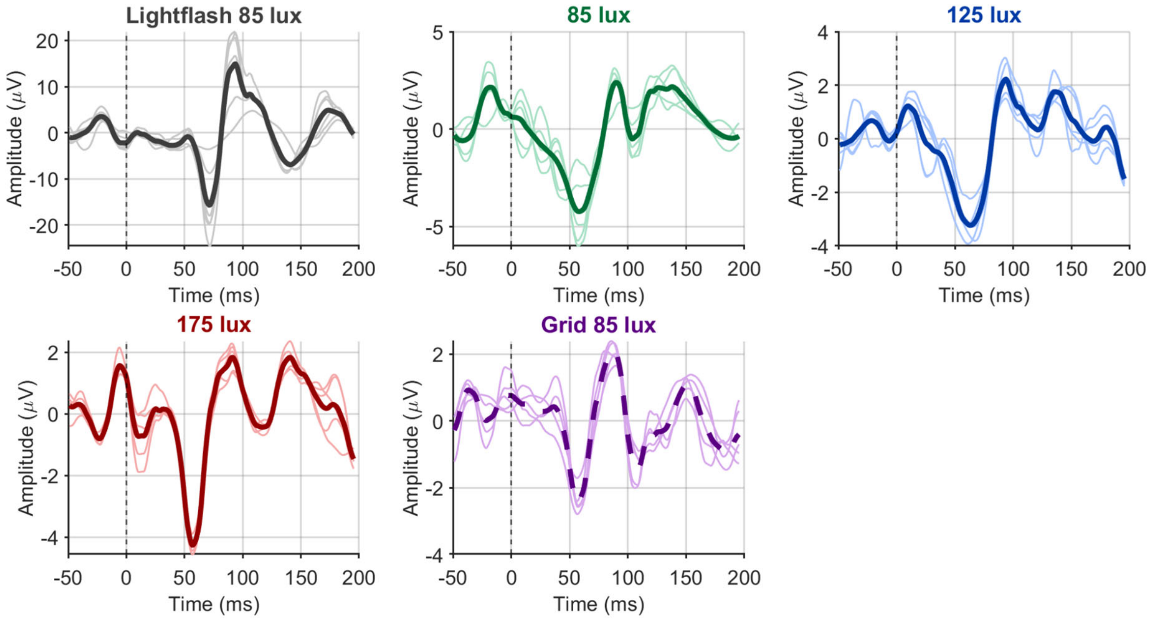

This figure demonstrates the stability of VEP waveforms using a sliding window analysis over 6 consecutive time windows (80 s long, with a 20 s step). Panels include: (A) light flash at 85 lux, (B) Micro-LED simulation at 85 lux, (C) Micro-LED simulation at 125 lux, (D) Micro-LED simulation at 175 lux, and (E) grid pattern at 85 lux. For each panel, the darker lines represent the overall average across the entire time span, while the lighter lines show the average for each sliding window. This analysis demonstrates minimal variation between time periods, indicating stable VEP responses under all stimulus conditions.
